# Dimensionality of genomic information and performance of the Algorithm for Proven and Young for different livestock species

**DOI:** 10.1186/s12711-016-0261-6

**Published:** 2016-10-31

**Authors:** Ivan Pocrnic, Daniela A. L. Lourenco, Yutaka Masuda, Ignacy Misztal

**Affiliations:** Department of Animal and Dairy Science, University of Georgia, Athens, GA 30602 USA

## Abstract

**Background:**

A genomic relationship matrix (GRM) can be inverted efficiently with the Algorithm for Proven and Young (APY) through recursion on a small number of core animals. The number of core animals is theoretically linked to effective population size (*N*_*e*_). In a simulation study, the optimal number of core animals was equal to the number of largest eigenvalues of GRM that explained 98% of its variation. The purpose of this study was to find the optimal number of core animals and estimate *N*_*e*_ for different species.

**Methods:**

Datasets included phenotypes, pedigrees, and genotypes for populations of Holstein, Jersey, and Angus cattle, pigs, and broiler chickens. The number of genotyped animals varied from 15,000 for broiler chickens to 77,000 for Holsteins, and the number of single-nucleotide polymorphisms used for genomic prediction varied from 37,000 to 61,000. Eigenvalue decomposition of the GRM for each population determined numbers of largest eigenvalues corresponding to 90, 95, 98, and 99% of variation.

**Results:**

The number of eigenvalues corresponding to 90% (98%) of variation was 4527 (14,026) for Holstein, 3325 (11,500) for Jersey, 3654 (10,605) for Angus, 1239 (4103) for pig, and 1655 (4171) for broiler chicken. Each trait in each species was analyzed using the APY inverse of the GRM with randomly selected core animals, and their number was equal to the number of largest eigenvalues. Realized accuracies peaked with the number of core animals corresponding to 98% of variation for Holstein and Jersey and closer to 99% for other breed/species. *N*_*e*_ was estimated based on comparisons of eigenvalue decomposition in a simulation study. Assuming a genome length of 30 Morgan, *N*_*e*_ was equal to 149 for Holsteins, 101 for Jerseys, 113 for Angus, 32 for pigs, and 44 for broilers.

**Conclusions:**

Eigenvalue profiles of GRM for common species are similar to those in simulation studies although they are affected by number of genotyped animals and genotyping quality. For all investigated species, the APY required less than 15,000 core animals. Realized accuracies were equal or greater with the APY inverse than with regular inversion. Eigenvalue analysis of GRM can provide a realistic estimate of *N*_*e*_.

## Background

Genomic best linear unbiased prediction (GBLUP) methods [1] for genomic evaluation use single-nucleotide polymorphism (SNP) effects indirectly via the genomic relationship matrix (GRM). Therefore, GBLUP-based methods require a GRM inverse, which has a cubic cost and can be computed efficiently for perhaps up to 150,000 individuals. Because of widely available commercial genotyping tools, some populations such as the U.S. Holstein cattle have over one million genotyped animals, and computing a GRM inverse can be prohibitively expensive. In addition, a GRM often is not positive definite, and additional steps (e.g., blending with a numerator relationship matrix) are required to make the GRM positive definite [1]. Misztal et al. [2] suggested an efficient computation of the GRM inverse by using recursion on a small subset of animals. Initially, this subset of animals was labeled as high accuracy or “proven”; therefore, the method was named the Algorithm for Proven and Young (APY). In this paper, we will refer to the GRM inverse calculated with this algorithm as the APY inverse and animals in the small subset as core animals. Compared with the regular GRM inverse, computing costs for the APY inverse are cubic only for the core subset and are linear for animals that are not in the subset. The estimated optimal subset size was approximately 8000 for Angus cattle [3] and 2000 to 6000 for commercial pigs [4]. Using U.S. Holstein data with 100,000 genotyped animals, Fragomeni et al. [5] found that any subset (including only bulls, only cows, and random animals) with at least 10,000 animals resulted in an accurate inverse. The APY inverse was successfully computed for about 570,000 genotyped Holsteins in less than 2 h of computing time on an average server with fewer than 20,000 core animals [6]. Using more than 10,000 animals as the core subset did not add any improvement in genetic prediction. For comparison, a regular inverse for 570,000 individuals would require several weeks of computing time and an amount of memory, which is available only in the largest computing clusters.

The theoretical framework of the APY inverse was proposed by Misztal [7]. For a population, the additive information is assumed to be in a limited number (*n*) of independent chromosome segments (*M*_*e*_) or effective SNP markers (ESM). If *M*_*e*_ or ESM completely explain the additive variation, the breeding values of *n* animals are linear functions of *M*_*e*_ or ESM and contain nearly all the information in *M*_*e*_ or ESM. Defining any subset of n animals as core animals, a recursion on any n animals is sufficient. The magnitude of *M*_*e*_ is a function of effective population size (*N*_*e*_), but the number of ESM could be computed as the number of eigenvalues explaining nearly all the variation in the GRM. Subsequently, the optimal number of core animals is a function of *N*_*e*_ and can be derived from eigenvalue analysis of the GRM.

The theory for APY inverse was tested by Pocrnic et al. [8] using six simulated populations with *N*_*e*_ ranging from 20 to 200. Each simulated population consisted of 10 non-overlapping generations under random mating and without selection, with 25,000 animals per generation and phenotypes available for generations 1 through 9. The last three generations (8 through 10) were completely genotyped, with 75,000 genotyped animals for each population. Their simulation assumed a total genome length of 30 Morgan and approximately 50,000 evenly allocated biallelic SNPs. They found that the number of largest eigenvalues that explain at least 90% of the variation in the GRM is almost a linear function of *N*_*e*_. For the number of largest eigenvalues that explain from 95 to 99% of the variation, the curve was curvilinear, with departure from linearity attributed to a limited number of SNPs and a limited number of genotyped animals. True accuracies were highest when the number of core animals corresponded to the number of eigenvalues explaining 98% of the variation, and they were slightly lower with the regular inverse or with half of the number of core animals.

The purpose of this study was to determine whether APY conclusions based on simulated data are valid with actual data across species. In particular, we wanted to find the optimal number of core animals per species, to investigate the changes in accuracy when recursions in APY are based on fractions of the optimal number of core animals, and to approximate the *N*_*e*_ for each species.

## Methods

### Data and models

Five previously collected datasets were used in this study. Analyses included the same models as those routinely used for national or commercial genetic evaluations of dairy (Holstein, Jersey) and beef (Angus) cattle, pigs, and broilers. The datasets and models were described in earlier studies [3, 6, 9–11]. Data for 11,626,576 Holstein final score records from 7093,380 cows were provided by Holstein Association USA, Inc. (Brattleboro, VT). Production data for Jerseys consisted of 4,168,048 records for 305-day milk, fat, and protein yields and were provided by the Animal Genomics and Improvement Laboratory, Agricultural Research Service, USDA (Beltsville, MD). For Angus cattle, more than 6 million records for birth weight and weaning weight and almost 3.4 million records for post-weaning gain were provided by the American Angus Association (St. Joseph, MO). More than 400,000 pig records for litter size and number of stillborn were provided by PIC (a Genus company, Hendersonville, TN). Finally, 196,613 records for body weight at grading, 51,774 records for residual feed intake, 9778 records for breast meat percentage, and 52,102 records for weight gain during feed conversion test were provided for broiler chickens by Cobb-Vantress Inc. (Siloam Springs, AR). The number of pedigrees used in the numerator relationship matrix (**A**) varied: 198,915 for broiler chickens, 2,429,392 for pigs, 2,468,914 for Jerseys, 8,236,425 for Angus, and 10,710,380 for Holsteins. The number of single-nucleotide polymorphisms used for genomic prediction and number of genotyped animals also varied: 60,671 SNPs for Jerseys and Holsteins with 75,033 and 77,066 genotyped animals, respectively; 38,321 SNPS and 80,933 genotyped Angus; 36,551 SNPs and 22,575 genotyped pigs; and 39,102 SNPs and 15,720 genotyped broiler chickens.

### Computations

Computations were similar to those described by Pocrnic et al. [8] except for the use of actual datasets and different validation strategies. The initial GRM (**G**_0_) was created for each dataset by using the methodology of VanRaden [1] as ${\mathbf{G}}_{0} = {\mathbf{ZZ}}^{{\prime }} /2\Sigma p_{\text{j}} \left( {1 - p_{j} } \right)$ where **Z** is a centered matrix of gene content adjusted for gene frequencies and *p*_j_ is allele frequency *p* for marker *j*. The observed allele frequencies were calculated directly from the SNP data of the genotyped population. The number of largest eigenvalues for **G**_0_ that explained 90, 95, 98, or 99% of variation was calculated using the DSYEV subroutine in LAPACK [12]. To obtain a positive definite GRM (**G**), **A** was blended with **G**_0_ as ${\mathbf{G}} = w{\mathbf{G}}_{0} + \left( {1 - w} \right){\mathbf{A}}_{22} ,$ where *w* is a weight different for each breed/species ranging from 0.90 to 0.95, and **A**_22_ is the pedigree-based numerator relationship matrix for genotyped animals [1].

Single-step GBLUP was used for genomic evaluation, and analyses were performed with BLUP90IOD2 software [13] either with the regular (direct) inverse of the **G** matrix [14] or the APY inverse [2, 6]. If **G** was partitioned into blocks corresponding to core (c) and non-core (n) animals:

$${\mathbf{G}} = \left[ {\begin{array}{*{20}c} {{\mathbf{G}}_{\text{cc}} } & {{\mathbf{G}}_{\text{cn}} } \\ {{\mathbf{G}}_{\text{nc}} } & {{\mathbf{G}}_{\text{nn}} } \\ \end{array} } \right],$$

then the APY inverse [2, 7] was:

$${\mathbf{G}}_{\text{APY}}^{ - 1} = \left[ {\begin{array}{ll} {{\mathbf{G}}_{\text{cc}}^{ - 1} } & 0 \\ 0 & 0 \\ \end{array} } \right] + \left[ {\begin{array}{ll} { - {\mathbf{G}}_{\text{cc}}^{ - 1} {\mathbf{G}}_{\text{cn}} } \\ \hspace{1.5em} {\mathbf{I}} \\ \end{array} } \right]{\mathbf{M}}_{\text{nn}}^{ - 1} \left[ { - {\mathbf{G}}_{\text{nc}} {\mathbf{G}}_{\text{cc}}^{ - 1} \quad {\mathbf{I}}} \right],$$

where ${\mathbf{M}}_{nn} = {\text{diag}}\left\{ {{\text{m}}_{{{\text{nn}},{\text{i}}}} } \right\} = {\text{diag}}\left\{ {{\text{g}}_{\text{ii}} - {\mathbf{g}}_{\text{ic}} {\mathbf{G}}_{\text{cc}}^{ - 1} {\mathbf{g}}_{\text{ci}} } \right\},$ ${\text{g}}_{\text{ii}}$ is the diagonal element of **G**_nn_ for non-core animal *i*, and ${\mathbf{g}}_{\text{ic}}$ is a vector of the genomic relationships of non-core animal *i* with all core animals. The number of core animals varied across datasets and corresponded to the number of largest eigenvalues in **G**_0_ that explained 90, 95, 98, or 99% of retained variation. The computational details for this algorithm were described by Masuda et al. [6].

### Validation

The validation method depended on the amount of information available for the animals. For Holsteins and Jerseys, daughter deviations [15] were calculated in the complete dataset without genomic information and used as the dependent variable. Genomic estimated breeding values (GEBV) were calculated based on truncated data and used as the independent variable in a linear regression model. The truncation point was defined by the year when the phenotype was recorded: 2009 for Holsteins and 2010 for Jerseys. Coefficient of determination (R^2^) for validation animals was used as a measure of reliability. For Holsteins, we defined the validation population as young genotyped bulls that had no daughters recorded in the truncated data, but had at least 30 daughters recorded in the complete dataset. For Jerseys, we defined the validation population as young genotyped bulls that had no daughters recorded in the truncated data, but had estimated breeding values (EBV) with at least 75% reliability in the complete data. The Holstein and Jersey validation populations included 2948 and 449 bulls, respectively.

For the other datasets, validation was done by predictive ability [16] based on correlations between GEBV and phenotypes adjusted for fixed effects. The Angus validation population consisted of 27,528 genotyped animals born in 2013 that had their phenotypes excluded from the truncated data. Among those 27,528 animals, 18,204 had phenotypes for body weight, 18,524 for weaning weight, and 10,471 for post-weaning gain. For pigs, the validation population consisted of 881 genotyped animals born in 2014 with repeated records for litter size and number of stillborn (1166 and 1229, respectively); their phenotypes were excluded from the truncated data. The broiler validation population consisted of 2975 genotyped birds from the last generation that had their phenotypes excluded from the truncated data. Among the validation birds, 2975 had records for body weight at grading, 1954 for residual feed intake, 215 for breast meat percentage, and 1964 for weight gain during feed conversion test.

Validation parameters (reliability or predictive ability) were computed for genomic evaluations that used the APY inverse with the corresponding number of randomly chosen core animals based on eigenvalues that explained 90–99% of original variation. Validation parameters were computed similarly for genomic evaluations that used the regular inverse of **G**.

## Results and discussion

Numbers of largest eigenvalues that explain 90, 95, 98, and 99% of variation in **G**_0_ are in Table 1 by breed/species. Number of eigenvalues that accounted for 90% of the original variation ranged from 1239 for pigs to 4527 for Holsteins, and those that accounted for 99% ranged from 5570 for broiler chickens to 19,397 for Holsteins. For each population, the total number of positive eigenvalues in **G**_0_ is limited by the number of SNPs and the number of genotyped animals.

**Table 1:** Numbers of largest eigenvalues that explain a given percentage of variation and estimated effective population size (*N*_*e*_)

Population	Number of genotyped animals	Number of SNPs	90%	95%	98%	99%	N_e_
Broiler chicken	15,720	39,102	1655	2606	4171	5570	44^a^
Pig	22,575	36,551	1239	2183	4103	6083	32^a^ (48)^b^
Angus cattle	80,993	38,321	3654	6166	10,605	14,555	113^a^
Jersey cattle	75,053	60,671	3325	6074	11,500	16,645	101^a^
Holstein cattle	77,066	60,671	4527	7981	14,026	19,379	149^a^

^a^Based on chromosome length of 30 Morgan

^b^Based on chromosome length of 20 Morgan

The distributions of eigenvalues that we obtained here for Holstein, Jersey, and Angus cattle, broiler chicken, and pig datasets were compared with those reported by Pocrnic et al. [8] for populations with an *N*_*e*_ of 20, 40, 80, 120, and 160 from a simulation study. In both cases, when the number of eigenvalues was plotted on a logarithmic scale, the curves were nearly linear. The distribution of eigenvalues observed for the Holstein dataset was nearly identical to that reported for a simulated population with an *N*_*e*_ of 160. The distribution of eigenvalues for the Angus and Jersey datasets were quite similar and intermediate to those found for simulated populations with an *N*_*e*_ of 80 and 120. For the pig dataset, the distribution of eigenvalues was intermediate to those found for simulated populations with an *N*_*e*_ of 20 and 40. Finally, for the broiler chicken dataset, the number of eigenvalues that explain 90% of the variation was close to that observed for a simulated population with an *N*_*e*_ of 40. As the proportion of explained variation increased, the number of eigenvalues for the broiler chicken decreased relative to those found for a simulated population with an *N*_*e*_ of 40. In general, the rank of the GRM was equal to or less than the number of genotyped animals and the number of SNPs. Smaller numbers of eigenvalues for the higher percentages of explained variation for the pig and broiler chicken datasets were likely the result of fewer genotyped animals (22,575 pigs and 15,720 broiler chickens) compared with the simulated population (75,000), since the rank of the GRM cannot exceed, and is likely smaller than, the number of genotyped animals. Another possible explanation is that fewer SNPs were used (36,000 for pigs and 39,000 for broiler chickens) compared with the 50,000 SNPs used in the simulation. MacLeod et al. [17] reported that the identification of 90% of the ancestral junctions between chromosome segments required 12 times as many SNPs as the number of junctions. Therefore, the number of chromosome segments that is determined by eigenvalue analysis will be underestimated if the number of SNPs (and genotyped animals) is too small. This may be generalized into a simple rule: the number of largest eigenvalues explaining a given percentage of variation is noticeably smaller than expected unless the corresponding number of SNPs (and perhaps genotyped animals) is at least 12 times larger. This condition was fulfilled when 90% of the variation was explained for all breeds/species but not when this percentage was higher.

**Fig. 1:**
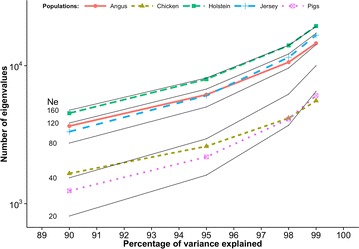
Numbers of largest eigenvalues explaining a given percentage of original variation. For broiler chickens, pigs, Angus, Jersey, Holstein cattle and simulated populations (*solid lines*) with different effective population sizes (*N*_*e*_ = 20, 40, 80, 120, 160). Simulated data were reported by Pocrnic et al. [8]

Assuming that the number of eigenvalues for 90% of explained variation was the least affected by the limited number of genotyped individuals and SNPs, *N*_*e*_ can be estimated by interpolation of real to simulated data (Fig. 1) at 90% of explained variation. Thus, estimated *N*_*e*_ were 149 for the Holstein, 113 for the Angus, 101 for the Jersey, 44 for the broiler chicken, and 32 for the pig populations (Table 1). Estimates of *N*_*e*_ based on genotypic information can be influenced by several factors. First, the estimates can be affected by genotype imputation because most of the animals are genotyped with lower density chips and their genotypes are then imputed to higher density (sometimes with multiple imputations). The final number of SNPs used for evaluation, the quality control of genomic data, and the length of the genome can vary by breed and species. The simulation study reported by Pocrnic et al. [8] assumed a genome length of 30 Morgan, which is appropriate for many species including cattle and broiler chickens [18–21]. Estimates of the genome length for pigs are consistently lower and range from 18 to 23 Morgan [22–25]. Assuming a genome length of 20 Morgan for pigs, the *N*_*e*_ would be 50% larger than that estimated from the simulated population since $N_{e} \sim1/{\text{L}}$ at a constant *M*_*e*_, where L is genome length in Morgan. Therefore, assuming a genome length of 20 Morgan, estimated *N*_*e*_ for pigs in our study would be 48. Many other factors including different recombination rates, different genome lengths for each sex and different genotyping patterns for each sex can influence the estimated *N*_*e*_. The assumptions in the simulations reported in [8] were idealistic in terms of population genetics (non-overlapping generations, random mating, no selection, and no migration), and differences in *N*_*e*_ resulted only from variation in sex ratios.

In the literature, estimates of *N*_*e*_ vary widely, and several approaches to calculate *N*_*e*_ have been reported (e.g., [26–28]). Leroy et al. [29] demonstrated variation in *N*_*e*_ estimates using different approaches. For Holsteins, *N*_*e*_ estimates range from 50 [30] to 150 [31], with many intermediate estimates in between [32–35]. Estimates for Jerseys range from 73 [34] to 135 [33]. For Angus, the *N*_*e*_ estimates vary from 26 [36] to 207 [37]. For various breeds of pigs, estimates can be as small as 55 [38] to as large as 113 [39]. Although *N*_*e*_ estimates for Holsteins and Jerseys are likely to be similar worldwide because of international breeding that is partially facilitated by the availability of Interbull evaluations, *N*_*e*_ estimates for pigs and broilers can vary because of the specific breeding structure used by individual companies. However, if different breeding companies use similar breeding plans, their individual populations may have a similar *N*_*e*_. Eitan and Soller [40] found that broiler companies that led breeding programs independently experienced similar problems (e.g., skeletal problems, metabolic disorders, hatchability problems, etc.) at the same time, indicating similar breeding plans.

**Fig. 2:**
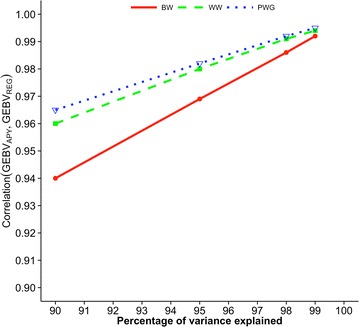
Correlations between GEBV_REG_ and GEBV_APY_ of validation animals for Angus cattle. Genomic estimated breeding values (GEBV) are based on the regular inverse (GEBV_REG_) and the Algorithm of Proven and Young inverse (GEBV_APY_) of the genomic relationship matrix. Traits are birth weight (BW), weaning weight (WW), and post-weaning gain (PWG). The number of core animals is defined as the number of eigenvalues that explain 90, 95, 98, and 99% of the original variation

**Fig. 3:**
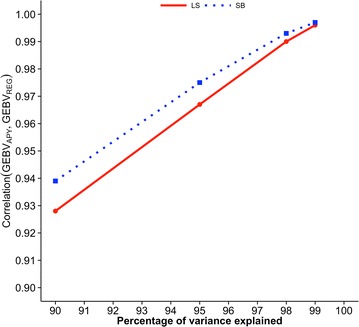
Correlations between GEBV_REG_ and GEBV_APY_ of validation animals for pigs. Genomic estimated breeding values (GEBV) are based on the regular inverse (GEBV_REG_) and the Algorithm of Proven and Young inverse (GEBV_APY_) of the genomic relationship matrix. Traits are litter size (LS) and number of stillborn (SB). The number of core animals is defined as the number of eigenvalues that explain 90, 95, 98, and 99% of the original variation

**Fig. 4:**
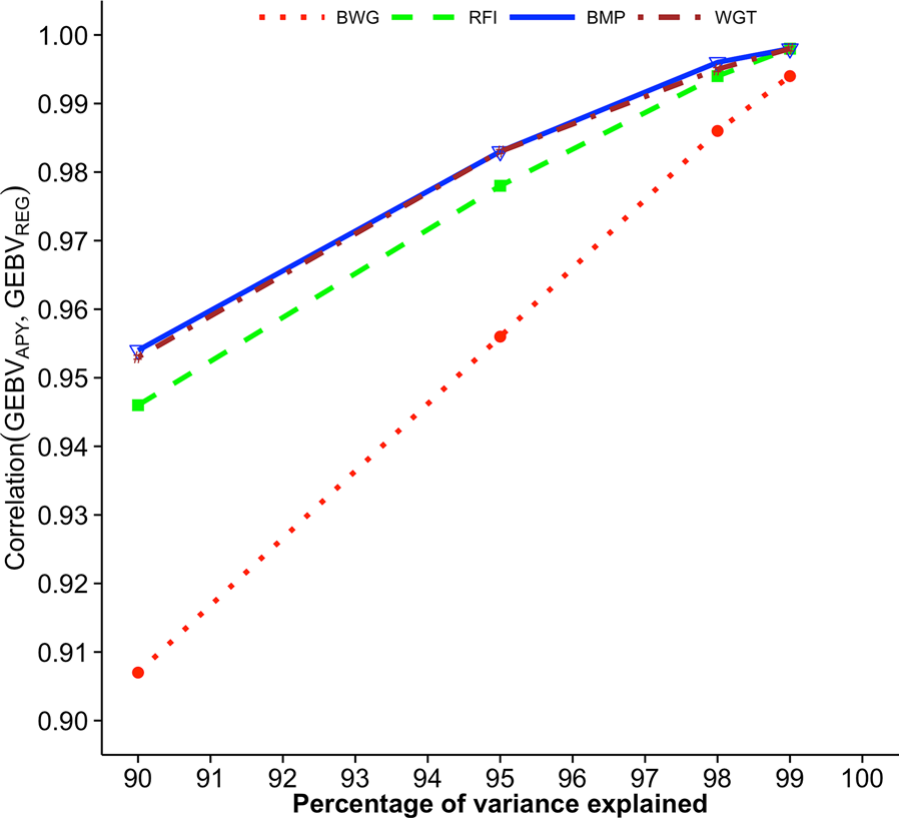
Correlations between GEBV_REG_ and GEBV_APY_ of validation animals for broiler chickens. Genomic estimated breeding values (GEBV) are based on the regular inverse (GEBV_REG_) and the Algorithm of Proven and Young inverse (GEBV_APY_) of the genomic relationship matrix. Traits are body weight at grading (BWG), residual feed intake (RFI), breast meat percentage (BMP), and weight gain during feed conversion test (WGT). The number of core animals is defined as the number of eigenvalues that explain 90, 95, 98, and 99% of the original variation

Figures 2, 3 and 4 show correlations between GEBV based on regular and APY inverses of **G** for Angus cattle, pig, and broiler chicken populations, respectively. These correlations are for validation animals that were obtained from the analysis with different numbers of core animals. For all species and traits, correlations were 0.99 when the number of core animals was equal to the number of largest eigenvalues of **G**_0_ that explained either 98 or 99% of the original variation. The linearity of the curves suggests that correlations between regular and APY GEBV are nearly a linear function of percentage of explained variation. Somewhat different slopes for different traits and breeds/species could be explained by the fact that GEBV for young animals are a weighted sum of parent average and direct genomic value with additional variation that depends on whether genotyped animals have genotyped parents [1, 11]. A smaller slope is usually observed for traits with a lower heritability because the weight on parent average is larger, does not depend on direct genomic value, and subsequently does not depend on the number of core animals.

**Fig. 5:**
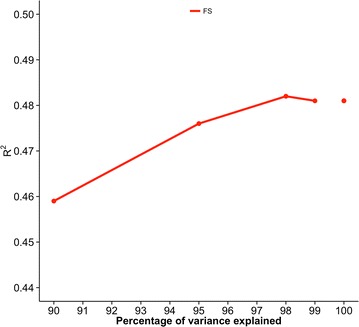
Coefficients of determination (R^2^) for final score (FS) of Holstein cattle. Value for 100% corresponds to the regular inverse of the genomic relationship matrix

**Fig. 6:**
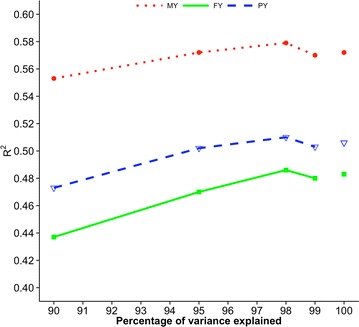
Coefficient of determination (R^2^) for 305-day milk yield (MY), fat yield (FY) and protein yield (PY) of Jersey cattle. Values for 100% correspond to the regular inverse of the genomic relationship matrix

**Fig. 7:**
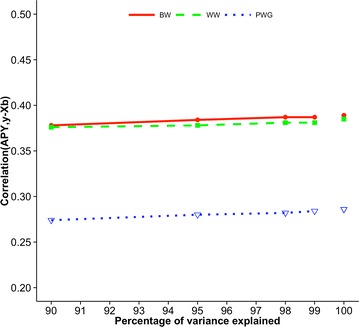
Predictive ability for birth weight (BW), weaning weight (WW), and post-weaning gain (PWG) of Angus cattle. Predictive ability is the correlation between genomic estimated breeding values based on the Algorithm of Proven and Young inverse of the genomic relationship matrix and phenotypes adjusted for fixed effects. The number of core animals is defined as the number of eigenvalues that explain 90, 95, 98, and 99% of the original variation; values for 100% correspond to the regular inverse of the genomic relationship matrix

**Fig. 8:**
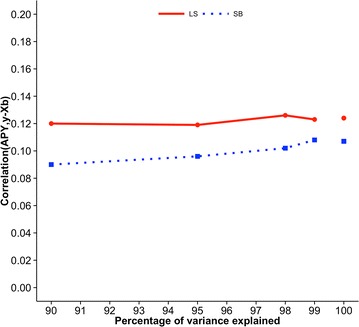
Predictive ability for litter size (LS) and number of stillborn (SB) of pigs. Predictive ability is the correlation between genomic estimated breeding values based on the Algorithm of Proven and Young inverse of the genomic relationship matrix and phenotypes adjusted for fixed effects. The number of core animals is defined as the number of eigenvalues that explain 90, 95, 98, and 99% of the original variation; values for 100% correspond to the regular inverse of the genomic relationship matrix

**Fig. 9:**
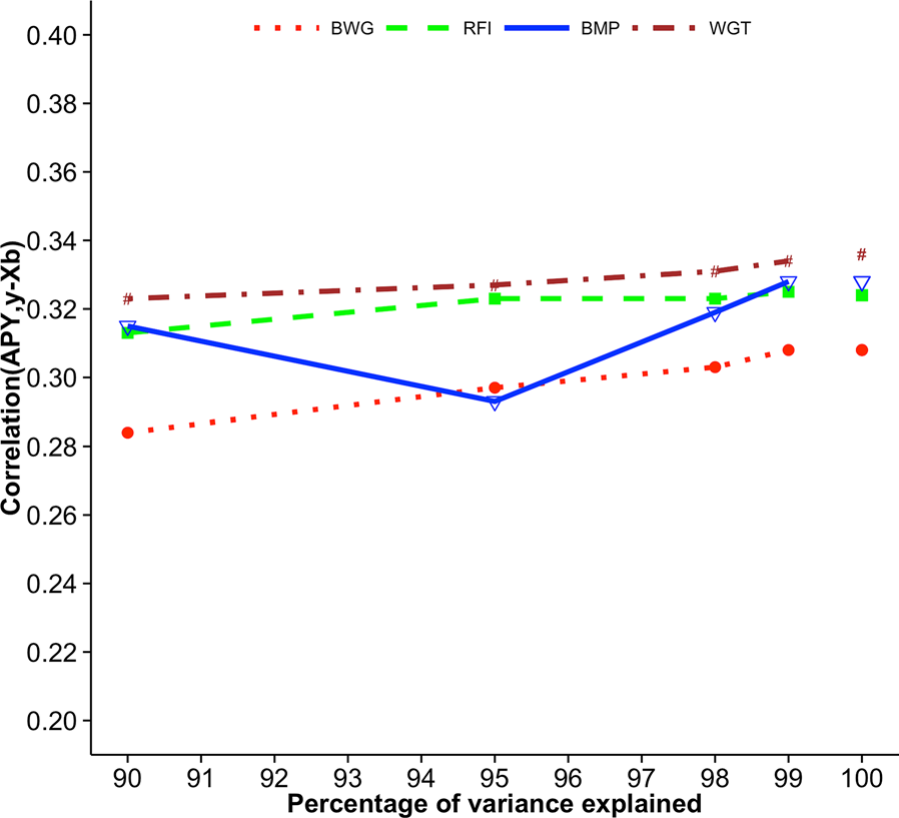
Predictive ability for body weight at grading (BWG), residual feed intake (RFI), breast meat percentage (BMP), and weight gain during feed conversion test (WGT) of broiler chickens. Predictive ability is the correlation between genomic estimated breeding values based on the Algorithm of Proven and Young inverse of the genomic relationship matrix and phenotypes adjusted for fixed effects. The number of core animals is defined as the number of eigenvalues that explain 90, 95, 98, and 99% of the original variation; values for 100% correspond to the regular inverse of the genomic relationship matrix

Figures 5, 6, 7, 8 and 9 show measures of accuracies as a function of the number of core animals: R^2^ for Holstein and Jersey cattle and predictive ability for Angus cattle, pigs, and broiler chickens. Realized accuracies (or reliabilities) were plotted as a function of the number of eigenvalues that explain a given percentage of variation, and values for 100% correspond to the regular inverse of the GRM. The highest accuracy for Holsteins and Jerseys (Figs. 5, 6, respectively) corresponded to 98% of explained variation as in the simulation study of Pocrnic et al. [8]. However, the curves for the remaining breed/species, which are based on predictive ability, were different. For Angus (Fig. 7), accuracy increased only slightly from 90 to 99% of explained variation. For pigs (Fig. 8), accuracy increases were again small, with almost no increase for litter size. For broilers (Fig. 9), the trend also was for small increases for all traits except breast meat percentage, which had an unexpected decrease at 95% of explained variation.

All flat trends occurred when accuracy was calculated based on predictive ability. Such accuracies are affected by model quality, especially the inclusion of less than optimal parameters in multiple-trait models. The flat trends and especially the anomalies can also be attributed to imputation issues as companies usually work with low- and medium-density SNP chips, which, in addition, are modified over the years.

An important question with the APY is whether the random choice of core animals as used in this study is optimal. In a Holstein study [9], the use of about 10,000 proven bulls plus their dams as core animals provided an increase in reliability of 0.01 over random choices. In a pig study [4], correlations of GEBV based on full and APY inverses were higher than 0.98 with a random sample of about 2000 core animals (10% sample) and higher than 0.99 with about 6000 core animals (20% sample); correlations were lower than 0.95 when using only the youngest or only the oldest generations as core animals. Breeding values of n animals in the core group are assumed to contain all the additive information about the population in terms of ESM or *M*_*e*_ [7]. For most complete information with as few animals as possible, the subset of animals should be representative of the population and (almost) linearly independent. These conditions seem to be fulfilled if choice is at random and clones are avoided. Ostersen et al. [4] reported marginally higher correlations of GEBV obtained with APY than with the regular inverse although higher correlations do not necessarily mean higher accuracy; the highest accuracy in a simulation [8] and partially in this study was obtained when these correlations were about 0.98–0.99.

Another question with the APY is whether the number and selection of core animals should change over time. In general, realized accuracy (reliability) was maximized when the number of randomly selected core animals was about 100 *N*_*e*_ or about 3 $N_{e} {\text{L}}$. That number is not critical since the accuracy (or reliability) decreased less than 0.01 when the number of core animals increased or was reduced by 50%. If breeding practices do not cause fast changes in *N*_*e*_ over generations, the same number of core animals selected randomly are likely to result in close to optimal evaluation accuracy. An exception could arise when the number of genotyped generations is large; under selection, older generations have little predictive power for selection candidates [41]. Further studies will determine whether the optimal approach in such a case is to choose core animals from younger generations or to remove old generations.

In this study, eigenvalue computations were done on an explicitly constructed **G**_0_, which actually shares the same eigenvalue distribution as the SNP BLUP matrix **Z'Z**. When large datasets are used, singular value decomposition of matrix **Z** can be applied instead, since it is equivalent to eigenvalue decomposition of **Z'Z** and **ZZ'** and to the eigenvalues of **G**_0_ multiplied by a constant. Therefore, the number of largest eigenvalues for **G**_0_ is identical between two quantities. Let the singular value decomposition of matrix **Z** be ${\mathbf{Z}} = {\mathbf{UDV}}'$, where **D** is a diagonal matrix of singular values that correspond to the square root of the non-zero eigenvalues of **Z'Z** and **ZZ'**. The columns of **U** are left singular vectors (${\mathbf{U'U}} = {\mathbf{UU'}} = {\mathbf{I}}$), and the columns of **V** are right singular vectors (${\mathbf{V'V}} = {\mathbf{VV'}} = {\mathbf{I}}$). They correspond to eigenvectors of **ZZ′** and ${\mathbf{Z}}^{{\prime }} {\mathbf{Z}}$, respectively. Then, ${\mathbf{Z}}^{{\prime }} {\mathbf{Z}} = {\mathbf{VD}}^{{\prime }} {\mathbf{U}}^{{\prime }} {\mathbf{UDV}}^{{\prime }} = {\mathbf{VD}}^{2} {\mathbf{V}}^{{\prime }}$, and $({\mathbf{Z}}^{'} {\mathbf{Z}}){\mathbf{V}} = {\mathbf{VD}}^{2}$, where **D** ^2^ is a diagonal matrix of eigenvalues of **Z'Z** (squares of singular values of matrix **Z**) and the columns of **V** are eigenvectors of **Z'Z**. Similarly, ${\mathbf{ZZ'}} = {\mathbf{UD}}^{2} {\mathbf{U'}}$. The singular value decomposition of **Z** can be computed using subroutine DGESVD in LAPACK [12], and computation cost will be quadratic for the number of markers but only linear for the number of individuals.

## Conclusions

The optimal number of core animals for efficient inversion of GRM by APY is about 14,000 for Holstein and Angus cattle, 12,000 for Jersey cattle, and 6000 for pigs and broiler chickens, which corresponds approximately to 3 $N_{e} {\text{L}}$. These numbers are not critical since reduction in GEBV accuracy is minimal if using half the optimal numbers. Approximate *N*_*e*_ with a genome length of 30 Morgan is 149 for Holsteins, 101 for Jerseys, 113 for Angus, and 44 for broiler chickens; for pigs and a genome length of 20 Morgan, approximate *N*_*e*_ is 48.
